# Compression Relaxation of Multi-Structure Polymer Composites in Penetrating Liquid Medium

**DOI:** 10.3390/polym14235177

**Published:** 2022-11-28

**Authors:** Alexander Kondratov, Valery Konyukhov, Stanislav Yamilinets, Ekaterina Marchenko, Gulsharat Baigonakova

**Affiliations:** 1 Department of Innovative Materials in the Print Media Industry, Moscow Polytechnic University, 107023 Moscow, Russia; 2 Department of Physical Chemistry, Mendeleev University of Chemical Technology, 125047 Moscow, Russia; 3 Laboratory of Superelastic Biointerfaces, National Research Tomsk State University, 634045 Tomsk, Russia

**Keywords:** compressive strain relaxation, polymer composites, polygraphy, offset printing blanket, liquid sorption, polymer swelling, diffusion coefficient, Flory–Huggins criterion

## Abstract

Multi-structural polymer composites are widely used in the mechanical engineering, automotive, aviation and oil refining industries, as well as in the printing industry as a shock-absorbing deckle on the offset cylinders of printing machines. During offset printing, composites come into contact with inks and washing solutions, the components of which penetrate the material and cause the polymers to swell. This process degrades the print quality, and for this reason the study of its features is relevant. The prerequisites for this work are the study of the fundamental laws of diffusion and sorption of liquids by polymers with different micro- and macro-structures in different physical states and in different forms (e.g., films, sheets, fibers and fabrics). The combination of polymer materials in the composition of multi-structural fabric blankets makes it possible to obtain materials with unique mechanical properties and high resistance to liquid penetrating media and to use them in high-tech processes of multi-color printing with high resolution and color rendering. This article reports for the first time the kinetics and thermodynamics results obtained from the swelling of multi-structural polymeric blankets in solvents used in printing, and the effect of sorption of different polar liquids on the viscoelastic strain under compression during the operation of the damping systems of printing machines. Using mathematical models of activated liquid diffusion in polymers and deformation of a viscoelastic body, the swelling rate constants, solvent diffusion coefficients (the kinetic characteristics of the swelling process) and Flory–Huggins parameters (the thermodynamic characteristics of the interaction of the solvent with the composite) for composite–solvent systems with several chemical composition variants were determined. The elastic modulus and the viscosity coefficient of the composite under liquid saturation were calculated based on the experimental cyclic compression data. The range of change in the compression and restoration times of the polymeric blankets (0.09 s ÷ 0.78 s) was determined. It was shown that the composite swelled to a limited extent in all the studied liquids. All solvents used were thermodynamically poor (χ > 0.5). It has been established that rubber–fabric blankets coated with nitrile rubber are the least resistant to the action of dichloroethane, and that blankets with layers of polyolefins are not resistant to ethyl acetate. Water significantly affects the physicochemical properties of rubber–fabric blankets with a large proportion of cotton fabric layers. The data obtained can serve as a basis for optimizing the compositions of inks and cleaning solutions, as well as a theoretical basis for the thermodynamics of composite–solvent systems.

## 1. Introduction

The compositions of the most common and effective damping materials used in mechanical engineering include elastic polymers and strong fibrous fillers [1,2,3,4,5]. The combination of polymers and high-strength fibers in the composition of multi-structural composites makes it possible to obtain materials with unique mechanical properties and high resistance to aggressive media. Many experimental and theoretical studies have been devoted to the study and prediction of changes in the mechanical properties of composites containing fibrous filler as a result of the sorption of water, aqueous solutions, and various organic liquids [1,2,3]. Rubber–fabric composites, which are multilayer blankets that transfer ink from the printing form to the printed material, are used in the printing industry. The most common composites are offset rubber–fabric blankets (ORFB) with a monolithic coating of butadiene-nitrile rubber (NBR) and ethylene propylene rubber (EPDM), as well as their mixtures (hybrids). Offset printing rubber blankets are produced by many international companies: FlintGroup (USA), TrelleborgCoatedSystems (Italy), ShanghaiDenghongMechanical (China) and others. The description of the trademarks of these materials must indicate the following: purpose, type of printing equipment, number of fabric layers, roughness, rigidity of the upper working layer and composite material as a whole, thickness, residual strain under compression and tension, relative elongation, tensile strength and compatibility with known inks and varnishes containing organic solvents or water [6]. The composite web is repeatedly deformed in constant contact with printing inks, washes and other process liquids, which accelerates the capillary penetration and sorption of liquids by all layers of the composite and leads to an increase in their size [7,8]. An increase in the size and change in the structure of the layers causes a deterioration in printing as well as a decrease in the mechanical and other operational characteristics of the canvases. Therefore, this study is relevant and there is a demand for the technology and its application [9,10].

The aim of this work is to quantitatively describe the compression strain hysteresis of a multilayer composite when impregnated with water and organic liquids which exhibit different thermodynamic affinity to composites containing polymer layers (monolithic coating, fabric and cellular foam) in order to predict the optimal pressure in the contact zones of the printing cylinders to ensure high printing quality.

Previously, the swelling of monolithic polymer plates (printing plates which are widely used in the flexographic printing method) in solvents of various chemical compositions was studied, followed by a mathematical analysis of the sorption kinetics [11,12,13,14]. 

The kinetic curve of the sorption of liquids by the composite in units of mass makes it possible to determine the swelling rate constant k and the coefficient of diffusion of the liquid into the composite D [11]. Analysis of the thermodynamics of the liquid–polymer interaction enables the calculation of the change in the Gibbs energy during blanket swelling Δ*G* or the Flory–Huggins parameter for a multicomponent polymer system χ [14,15].

Understanding the relationship between the sorption processes occurring in composites in contact with liquids, stress [16] and ORFB deformation kinetics as well as the reason for the loss of the elastic properties of polymer composites is important for various applications [16,17,18] and for the uninterrupted operation of offset printing machines [18]. 

## 2. Materials and Methods

The effect of solvents with different molecular polarities and thermodynamic properties on the following composite sheets of the Heidelberg-CIS company FlintGroupDayGraphica (UK) was studied: Saphira 1000 (printed layer of the fabric—ethylene-propylene rubber—“EPDM”), 3610 (printed layer of the fabric—butadiene-nitrile rubber—“NBR”), Explorer (NBR), 0047 (the printed layer of the web is a mixture of nitrile butadiene and ethylene-propylene rubber—“Hybrid”).

**Figure 1:**
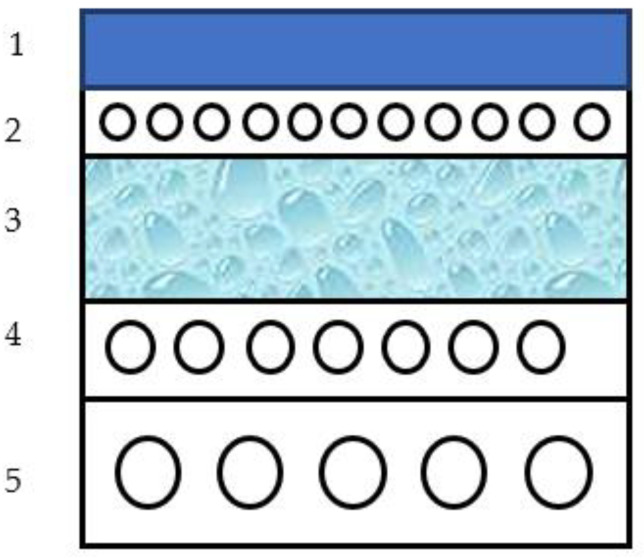
The structure of the multilayer rubber–fabric composite used for offset printing. 1—ink-transferring monolithic layer, 2,4,5—fabric layers, 3—foamed elastomer layer.

The polymer composite layers are located on the upper ink-transferring surface and inside the blanket; the fabric layers are inside and on the underside of the blanket (Figure 1). The geometric proportion of fabrics in the thickness of blankets varies for different rubber–fabric composite brands: Saphira 1000 (60%), 3610 (55%), Explorer (70%), 0047 (70%). The reinforced woven fabrics of the layer composites consist of calibrated purified cotton fabric, which is 98% pure cellulose (Figure 1).

To quantify the effect of a penetrating liquid medium on the compression relaxation parameters of composites containing different numbers of cotton fabric hydrophilic layers in the multi-structural polymeric blankets, this article compares samples with the same external monolithic layer made of nitrile butadiene rubber: Explorer (NBR) and 3610 (NBR). 

Using the IR Fourier spectroscopy method of printing inks and technological solutions, their main ingredients were established. Based on the chemical structure and physicochemical properties of the detected ingredients, six model liquids of different polarity were selected, each having a chemical structure similar to their elemental composition and chemical structure.

The physical and chemical resistance of blankets to aggressive environments of different polarities were experimentally evaluated using analytical-grade liquids as models of solvents and process solutions (Table 1).

Samples were cut from the blankets in the form of squares with 30-mm sides. The samples were weighed with an accuracy of 0.005 g and the thickness was measured with an accuracy of 0.01 mm.

The samples were placed in a sealed box filled with the solvent under study and completely immersed in the solvent. The samples were periodically removed, cleaned of excess solvent with filter paper, and weighed in a closed box using the analytical balance with an error of 0.001 g.

After attaining the sorption equilibrium, the linear dimensions of the extremely swollen samples were measured using a TIB-1 thickness gauge in accordance with GOST. The swelling of all the studied composites ended within a few minutes and further contact with the solvent did not change the deformation and thermodynamic properties of the system.

The deformation of the samples under the action of a fixed constant stress (0.8 MPa) was measured using a modified thickness gauge [12] and using a laboratory stand with stress sensors [19]. The elastic modulus E was calculated according to ISO 7743-2013.

To assess the strain relaxation of the ORFB, the initial and extremely swollen samples were compressed using a TIB-1 thickness gauge under a load of 0.8 MPa for 5 seconds (t_5_), after which the load was removed and the blanket recovery was recorded within 5 seconds (t_10_). The entire process of the changing of the thickness was continuously recorded using video recording tools, followed by the framing and establishment of changes in the structure and thickness of the composite according to the methods [12,20].

## 3. Results and Discussion

Swelling of multi-structural polymer blankets results from the following simultaneous processes: capillary penetration of the solvent between the layers and fabric fibers, solvent diffusion into the polymer coating, volume strain during swelling of the polymer matrix, solvent diffusion into polymer fibers and strain during swelling of the fabric fibers.

Liquid diffusion into polymers depends on the thermodynamics of their interaction, and its intensity is determined by the Flory–Huggins parameter χ. The thermodynamic affinity that causes the swelling and dissolution of polymers in liquids equals χ < 0.5 [21].

To calculate the diffusion coefficient D of a liquid in polymers [22] from a change in the thickness of a prismatic sample of infinite length, the following equation can be used:

(1) $$-\ln\left(1-\frac{m\left(t\right)}{m_{E}}\right)=a_{1}^{2}\times h^{-2}\times D\times t,$$

where m(t) is the mass of the liquid absorbed by the sample within time t, $m_{E}$ is the limiting value of the mass of the liquid absorbed by the sample, the proportionality coefficient $a_{1}$ is calculated according to the method proposed in [22]. $\Phi_{E}$ is the volume fraction of the polymer in its equilibrium state:

(2) $$\Phi_{E}=\frac{W_{0}}{W_{1}},$$

where W_0_ is the initial volume (mm^3^), and W_1_ is the final volume (mm^3^).

The parameter χ can be calculated using the Flory–Rener equation [16,20,23]:

(3) $$\ln\left(1-\Phi_{E}\right)+\Phi_{E}+\chi \times \Phi_{E}^{2}+Z^{-1}\times \Phi_{E}^{\frac{1}{3}}=0,$$

(4) $$\chi =-\frac{\ln\left(1-\Phi_{E}\right)+\Phi_{E}+Z^{-1}\times \Phi_{E}^{\frac{1}{3}}}{\Phi_{E}^{2}},$$

(5) $$Z=\frac{V_{2}}{V_{1}},$$

where Z is a dimensionless parameter equal to the ratio of the molar volume of the subchains of the polymer network $V_{2}$ to the molar volume of the solvent $V_{1}$ [22]:

(6) $$V_{2}=R\times T\times E^{-1}\times \phi^{\frac{1}{3}},$$

where R is the universal gas constant, E is the elasticity modulus of the swollen polymer and ϕ is the local volume fraction of the polymer in the polymer–solvent system.

All the constants of equation 1 are transferred to obtain the dependence of the mass of the liquid absorbed by the sample on the contact time f(t).

(7) −ln(1−g(t))=f(t),

where g is the ratio of the mass of the liquid absorbed by the sample to the limit mass of the liquid absorbed by the sample, and the diffusion coefficient D and cm^2^/s of liquid penetrating into the composite is determined from the slope of the straight line. In this case, the swelling rate constant k can be calculated using the following equation:

(8) $$\ln(\frac{a_{\infty }}{a_{\infty }-a_{t}})=k\times t\;,$$

where $a_{t}$ is the degree of composite swelling equal to the ratio of the mass of the liquid absorbed during time t to the initial mass of the sample and $a_{\infty }$ is the maximum degree of swelling of the extremely swollen sample equal to the ratio of the maximum mass of the absorbed liquid to the initial mass of the sample.

The tangent of the angle of the inclined straight line in these coordinates will correspond to the swelling rate constant k, min^−1^ [12].

The physical parameters of a layered rubber–fabric composite and its ability to recover under high-frequency compression can be described using the Kelvin–Voigt viscoelastic body model. The model consists of parallel connected elements: elastic (with elastic modulus E) and viscous (with viscosity coefficient η). The relaxation time of this system is expressed by the equation [24,25]:

(9) $$\tau =\frac{\eta }{E},$$

Deformation of the model in the processes of compression and recovery [26] is described by the following equations. For the material compression process:

(10) $$\gamma =\frac{\sigma }{E}\times \left(1-e^{-\frac{E}{\eta }\times t}\right),$$

and for material recovery process:

(11) $$\gamma =\frac{\sigma }{E}\times e^{-\frac{E}{\eta }\times t},$$

where γ is the normalized value of changes in the system linear dimensions, i.e., relative strain:

(12) $$\gamma =\frac{l_{0}-l_{t}}{l_{0}},$$

where $l_{0}$ and $l_{t}$ are, respectively, the sample thickness before mechanical exposure and the sample thickness at the moment of time variation t (mm).

Equation (10) for the compression process of the composite sample can be rewritten as follows:

(13) $$\gamma_{\infty }-\gamma =\frac{\sigma }{E}\times e^{-\frac{E}{\eta }\times t},$$

where $\gamma_{\infty }=\frac{\sigma }{E}$ and $\Delta \gamma =(\gamma_{\infty }-\;\gamma )$— are the differences in relative strains. If we construct the function Δγ=f(t) to find $\Delta \gamma_{0}={\;\gamma }_{\infty }-0$ and $\Delta \gamma_{0}={\;\gamma }_{\infty }=\frac{\sigma }{E}$, we get $\Delta \gamma =\Delta \gamma_{0}\cdot e^{-\frac{E}{\eta }\cdot t}$ and, with regard to Equation (10), obtain:

(14) $$\Delta \gamma =\Delta \gamma_{0}\times e^{-\frac{1}{\tau }*t},$$

Logarithmation of (14) brings it to the linear form:

(15) $$\ln\left(\Delta \gamma \right)=\ln\left(\Delta \gamma_{0}\right)\times (-\frac{1}{\tau })\times t,$$

or another modification of Equation (15):

(16) $$\ln\left(\frac{\Delta \gamma_{0}}{\Delta \gamma }\right)=\frac{1}{\tau }\times t\;,$$

can be used to determine the relaxation time of the blanket thickness during compression from the cotangent of the inclination angle of the line in the coordinates:

$$\ln\left(\frac{\Delta \gamma_{0}}{\Delta \gamma }\right)=f\left(t\right)$$

A similar consideration of the sample recovery allows the solving of the equation of the Kelvin–Voigt model in the following form:

(17) $$\ln\left(\frac{\gamma_{0}}{\gamma }\right)=\frac{1}{\tau }\times t,$$

The function $\ln\left(\frac{\gamma_{0}}{\gamma }\right)=f\left(t\right)$ is constructed to determine the relaxation time of the blanket thickness during recovery from the cotangent of the inclination angle of the line of the dependency graph $\ln\left(\frac{\Delta \gamma_{0}}{\Delta \gamma }\right)=f\left(t\right)$.

**Figure 2:**
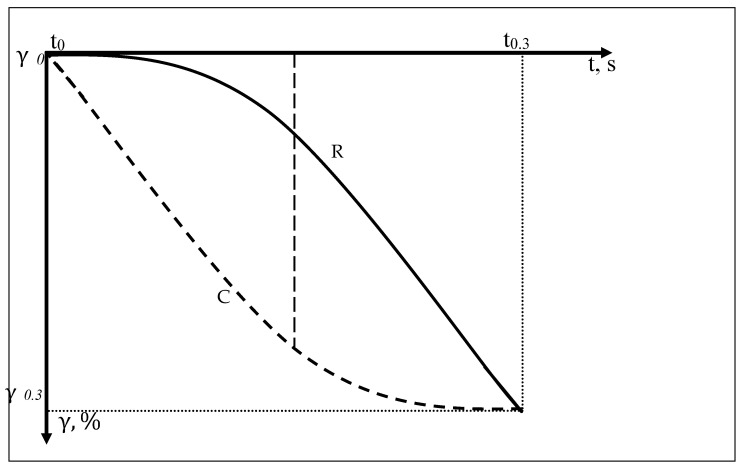
Change in the thickness of the composite sample under short-term cyclic compression loads. C is compression from 0 s $\left(t_{0}\right)$ to 0.3 s $\left(t_{0,3}\right)$, R is recovery from 0.3 s $\left(t_{0,3}\right)$ to 0.6 s $\left(t_{0}\right)$.

To quantify the change in the blanket thickness at the minimum cylinder speed (5 Hz), the strain hysteresis is plotted using the compression and recovery functions (9, 10) on the reverse time axis in the range of 0–0.3 s (Figure 2).

To assess the internal friction during compression and recovery of the layer blanket, the hysteresis parameter is used, which can be calculated using the following equation:

(18) $$K_{a}=\frac{\gamma_{↑}-\gamma_{↓}}{\gamma_{↑}}\times 100\%,$$

where $\gamma_{↓}$ is the relative strain in the lower half of the cycle, and $\gamma_{↑}$—is the relative strain in the upper half of the cycle.

The physical properties of the rubber–fabric composite layers changed under the impact of the contacting liquid during cyclic compression and were quantitatively described using the parameters of the Kelvin–Voigt viscoelastic model: strain relaxation time (τ), elastic modulus (E) and dynamic viscosity coefficient (η). The calculated values of these parameters are summarized in Table 2, Table 3, Table 4 and Table 5.

The sorption of liquids by the composites, expressed in mass units, was used to determine the swelling rate constant k, the diffusion coefficient of liquid penetrating into the composite D and the Flory–Huggins parameter χ.

These characteristics of mass transfer and thermodynamic affinity are presented in Table 6, Table 7, Table 8 and Table 9.

For an objective assessment of the dynamics of compression and recovery of a composite web under conditions of contact with various liquids and in air, one should use the structure-sensitive parameter of the Kelvin–Voigt model of a viscoelastic body—the relaxation time. The EPDM-coated web has a significant relaxation time under pressure compression at about 0.8 MPa (0.45 s). The relaxation time is two decimal orders longer than the compression time. This causes a strong resistance of the web during rapid deformation. Since the compression in the contact band lasts no more than 0.003 s, the load exerted on the web will also be significantly higher than the equilibrium value. This causes excessive dot gain of thin strokes and dotted features, such as letters and halftone dots, during printing and high pressure on the printed substrate. The compression pressure increases many times after the penetration of water and dichloroethane into the porous structure of the composite web through the layers of cotton fabric [25,26]. The relaxation time is 0.64 s and the compression time is 0.66 s. These liquids have close values of the dipole moments of the molecules.

In air, the restoration of the web after compression occurs three times faster than during compression and does not significantly depend on contact with organic solvents. Water ‘slows down’ the recovery of thickness even during its slight penetration into the blanket. The volume fraction of the polymer after contact with water is 0.97. The blanket swells to a limited extent in all the test liquids, since all of the solvents have poor thermodynamic properties (χ < 0.5).

In air, the 3610 (NBR) blanket exhibits half the compression relaxation time (0.22), which increases significantly after its contact with ethyl acetate and water. The rate of the relaxation processes does not change during the compression and recovery of the blanket, and the effect of liquids on its recovery is negligible. Even dichloroethane, which causes the maximum swelling of polymers (up to 140 wt %), does not significantly affect the rate of relaxation.

Blanket for convection printing. The composite shows high resistance to all solvents. The blanket swells to a limited extent (up to 140 wt%) in dichloroethane.

The Explorer brand (NBR) convection printing canvas has a higher fabric content than the 3610 brand, and a comparable proportion of rubber components. The web exhibits good resistance to all the solvents tested, except water, which significantly increases the recovery time of the web. The composite swells to a limited extent (up to 121 wt%) in dichloroethane.

The hybrid blanket has average values located between the corresponding parameter values of the blankets with mono elastomer compositions and allows printing and cleaning with almost all types of solutions. The hybrid blanket loses its recoverability in polar liquids (dichloroethane and water). Water increases the relaxation time of the blanket when it is compressed. The blanket swells to a limited extent (up to 105 wt %) in dichloroethane.

## 4. Conclusions

1. The kinetics and thermodynamics of composites swelling in solvents, which are chemical models of the liquid components of operational media, have been studied using shock-absorbing, multi-structural polymer composite blankets applied, for example, in printing technology. In this article, the rate constants and coefficients of solvent diffusion into multilayer composites were calculated. Flory–Huggins parameters χ of solvent–composite systems were determined. For all solvents, χ > 0.5, which corresponds to the limited thermodynamic compatibility of the components and ensures the insolubility and relative physicochemical stability of the composite in the test solvents, but does not guarantee the high quality of multi-color printing by the traditional offset method with continuous dampening of the printing plate.
2. The cyclic deformation of the Kelvin–Voigt viscoelastic model was used to quantitatively describe the compression and recovery of the geometric dimensions of swollen composites of different composition after cyclic loading. The compression times and the recovery times of the blankets (0.09 s ÷ 0.78 s) were determined to compare the effects of solvents of different thermodynamic quality on the studied rubber–fabric composites in terms of the Flory–Huggins value χ and to predict their behavior under cyclic compression.
3. It has been established that composite rubber–fabric blankets coated with nitrile butadiene rubber (NBR) are least resistant to dichloroethane, and that blankets with ethylene propylene diene monomer (EPDM) layers are not resistant to ethyl acetate. Water significantly affects the physicochemical properties of rubber–fabric blankets with a large proportion of cotton fabric layers. For the 3610 (NBR) blanket with an NBR outer layer, the relaxation time increases from 0.2 s to 0.46 s, while for the Explorer (NBR) blanket it decreases from 0.2 s to 0.09 s. This multidirectional effect of the penetrating liquid medium on the blankets’ compression relaxation is due to the different content of the hydrophilic layers of cotton fabric in the structure of the multi-structural polymer composites.

## Figures and Tables

**Table 1:** Elemental composition, designation and polarity of the studied liquids molecules.

Liquid	Abbreviation or Molecular Formula	Dipole Moment, D
carbon tetrachloride	CCl_4_	0
toluene	C_7_H_8_	0.37
isopropyl alcohol	IPA	1.66
1,2-dichloroethane (ClCH₂CH₂Cl)	EDC	1.80
ethyl acetate	C_4_H_8_O_2_	1.81
water	H_2_O	1.86

**Table 2:** Physical properties of the ‘composite–liquid’ system for the Saphira 1000 (EPDM) blanket.

		Air	IPA	C_7_H_8_	C_4_H_8_O_2_	EDC	CCl_4_	H_2_O
τ, s	Compression	0.45	0.39	0.49	0.45	0.66	0.47	0.64
E, MPa		11.64	12.90	7.45	5.90	8.73	8.74	9.32
η, MPa·s		5.20	5.05	3.66	2.65	5.77	4.10	6.01
$\sigma_{\mathrm{cp}}$, MPa		0.87	0.97	0.56	0.44	0.65	0.66	0.70
τ, s	Recovery	0.16	0.14	0.20	0.15	0.13	0.18	0.38
E, MPa		8.71	10.05	10.06	1.32	4.94	9.96	25.97
η, MPa·s		1.40	1.37	1.99	0.16	0.62	1.75	9.82
$K_{a}$, %		95.00	95.48	90.14	98.51	94.56	92.03	73.00

**Table 3:** Physical properties of the ‘composite–liquid’ system for the 3610 (NBR) blanket.

		Air	IPA	C_7_H_8_	C_4_H_8_O_2_	EDC	CCl_4_	H_2_O
τ, s	Compression	0.22	0.31	0.33	0.57	0.25	0.23	0.45
E, MPa		17.20	4.90	3.44	5.48	2.98	3.41	5.82
$\sigma_{\mathrm{cp}}$, MPa		1.29	0.37	0.26	0.41	0.22	0.26	1.29
τ, s	Recovery	0.22	0.10	0.18	0.19	0.15	0.12	0.21
E, MPa		16.81	2.50	4.71	4.24	4.59	3.38	3.89
$K_{a}$, %		96.72	97.65	93.21	93.48	94.20	96.58	95.50

**Table 4:** Physical properties of the ‘composite–liquid’ system for the Explorer (NBR) blanket.

		Air	IPA	C_7_H_8_	C_4_H_8_O_2_	EDC	CCl_4_	H_2_O
τ, s	Compression	0.21	0.29	0.25	0.28	0.15	0.15	0.09
E, MPa		9.42	9.15	6.39	5.63	2.07	4.18	4.22
$\sigma_{\mathrm{cp}}$, MPa		0.69	0.48	0.42	0.16	0.31	0.32	0.69
τ, s	Recovery	0.16	0.14	0.21	0.13	0.13	0.17	0.38
E, MPa		9.90	9.73	9.29	1.19	4.49	8.73	26.0
$K_{a}$, %		96.66	95.38	94.48	99.11	95.08	95.23	91.25

**Table 5:** Physical properties of the ‘composite–liquid’ system for the 0047 (Hybrid) blanket.

		Air	IPA	C_7_H_8_	C_4_H_8_O_2_	EDC	CCl_4_	H_2_O
τ, s	Compression	0.23	0.15	0.12	0.38	0.24	0.16	0.78
E, MPa		4.73	12.14	3.27	9.46	3.43	3.36	8.83
η, MPa·s		1.09	1.82	0.392	3.59	0.823	0.538	6.89
$\sigma_{\mathrm{cp}}$, MPa		0.91	0.25	0.71	0.26	0.25	0.66	0.91
τ, s	Recovery	0.16	0.25	0.14	0.23	0.38	0.22	0.85
E, MPa		10.61	28.07	5.33	21.80	5.35	4.39	12.40
η, MPa·s		1.72	7.14	0.76	4.99	2.05	0.97	10.6
$K_{a}$, %		92.21	94.66	97.03	86.78	94.16	96.80	82.39

**Table 6:** Thermodynamic properties of the ‘composite–liquid’ system for the Saphira 1000 (EPDM) blanket.

	IPA	C_7_H_8_	C_4_H_8_O_2_	EDC	CCl_4_	H_2_O
k, min^–1^	0.0257	0.0300	0.0519	0.0406	0.0203	0.0667
D	0.0094	0.0236	0.0299	0.0429	0.0237	0.0251
$\alpha_{\infty }$, %	5.56	61.09	31.20	83.96	89.11	6.01
χ	2.43	1.06	1.42	1.03	1.05	2.88
$\Phi_{E}$	0.96	0.72	0.84	0.70	0.71	0.97

**Table 7:** Thermodynamic properties of the ‘composite–liquid’ system for the 3610 (NBR) blanket.

	IPA	C_7_H_8_	C_4_H_8_O_2_	EDC	CCl_4_	H_2_O
k, min^–1^	0.0122	0.0157	0.0234	0.0138	0.0129	0.0117
D	0.0044	0.0125	0.0153	0.0240	0.0112	0.0044
$\alpha_{\infty }$, %	11.16	64.14	49.59	139.14	70.55	10.06
χ	2.06	0.98	1.14	0.85	1.15	2.70
$\Phi_{E}$	0.93	0.67	0.75	0.57	0.76	0.97

**Table 8:** Thermodynamic properties of the ‘composite–liquid’ system for the Explorer (NBR) blanket.

	IPA	C_7_H_8_	C_4_H_8_O_2_	EDC	CCl_4_	H_2_O
k, min^–1^	0.0197	0.0216	0.0236	0.0192	0.0183	0.0132
D	0.0083	0.0170	0.0170	0.0312	0.0160	0.0054
$\alpha_{\infty }$, %	12.03	52.6	44.2	120.7	59.6	8.86
χ	1.87	1.09	1.25	0.90	1.24	2.85
$\Phi_{E}$	0.92	0.73	0.79	0.62	0.79	0.97

**Table 9:** Thermodynamic properties of the ‘composite–liquid’ system for the 0047 (Hybrid) blanket.

	IPA	C_7_H_8_	C_4_H_8_O_2_	EDC	CCl_4_	H_2_O
k, min^–1^	0.0119	0.0151	0.0305	0.1844	0.0112	0.0203
D	0.0048	0.0120	0.0206	0.2188	0.0086	0.0076
$\alpha_{\infty }$, %	10.88	57.25	47.65	104	53.49	7.84
χ	2.30	1.05	1.11	1.04	1.33	4.91
$\Phi_{E}$	0.95	0.71	0.74	0.70	0.82	1.0

## Data Availability

Not applicable.
